# Sociodemographic characteristics, attitudes, and knowledge associated with previous screening for cervical cancer among women in western Jamaica

**DOI:** 10.1186/s13027-023-00537-4

**Published:** 2023-09-23

**Authors:** Pauline E. Jolly, Anna Junkins, Maung Aung

**Affiliations:** 1 Department of Epidemiology, School of Public Health, University of Alabama at Birmingham, 1665 University Blvd, Birmingham, AL 35233 USA; 2 Epidemiology and Research Unit, Western Regional Health Authority, Montego Bay, Jamaica

**Keywords:** Cervical cancer, Screening uptake, Jamaica, Pap test, Predictors

## Abstract

**Background:**

About 90% of new cervical cancer cases and deaths worldwide in 2020 occurred in low- and middle-income countries. This can be attributed to the low rates of cervical cancer screening in these countries. This study was conducted to identify factors associated with lack of cervical cancer screening among women in western Jamaica with the aim to increase screening and decrease cervical cancer risk.

**Methods:**

This cross-sectional study assessed associations between previous Pap testing or lack of testing in five years or more, sociodemographic characteristics, attitudes, and knowledge of cervical cancer among women recruited from clinics and community events in the four parishes of western Jamaica. Analyses included chi-square tests, Fisher’s exact tests, and logistic regression.

**Results:**

Of the 223 women included in the study, 109 (48.9%) reported Pap testing five years or more previous to the study. In the multivariate analysis, women from St. James (Odds Ratio [OR]: 3.35, 95% Confidence Interval [CI]: 1.12–9.99), Trelawny (OR: 5.34, 95% CI: 1.23–23.25), and Westmoreland (OR: 3.70, 95% CI: 1.10–12.50) had increased odds of having had Pap test screening compared to women from Hanover. Women ≥ 50 years of age compared to women 18–29 years of age (OR: 6.17, 95% CI: 1.76–21.54), and employed compared to unemployed women (OR: 2.44, 95% CI: 1.15–5.20) had increased odds of Pap test screening. Similarly, women with one (OR: 4.15, 95% CI: 1.06–16.22) or two or more children (OR: 8.43, 95% CI: 2.24–31.63) compared to women with no children had higher odds of screening. Women who were aware, compared to women who were unaware, of the purpose of Pap tests had increased odds of screening (OR: 3.90, 95% CI: 1.55–9.82). Lastly, women who believed Pap tests were painful compared to women who did not, had decreased odds of having had a Pap test (OR: 0.33, 95% CI: 0.16–0.71).

**Conclusions:**

Uptake of Pap tests among the women was suboptimal and varied among parishes. Young women and women without children were less likely to have ever been screened. Increased education of the purpose of Pap tests to treat pre-cancer to prevent cancer and minimization of the notion that Pap tests are painful could promote screening among women in this population.

## Background

Cervical cancer is the fourth most diagnosed cancer among women worldwide [1, 2]. There were an estimated 604,000 new cases and 342,000 deaths caused by cervical cancer in 2020. Globally, the cervical cancer incidence and mortality rates are high, at an age standardized incidence rate of 7.3 per 100,000 person-years and age-standardized mortality rate of 13.3 per 100,000 person-years in 2020 [2]. The burden of cervical cancer falls disproportionately on low and middle-income countries (LMICs) compared to high-income countries. Furthermore, about all (90%) of cervical cancer deaths occur in LMICs [2]. In 2020, the incidence and mortality age-standardized rates for cervical cancer in the Caribbean were 13.7 and 8.2 per 100,000 person-years, respectively [1] which were more than twice the rates in North America (6.2 and 2.1 per 100,000 person-years, respectively). The burden of cervical cancer in Jamaica reflects this disparity. The estimated age-standardized incidence and mortality rates of cervical cancer in Jamaica in 2023 was 21.6 and 13.6 per 100,000 person years, respectively; these rates exceed both global and Caribbean estimates of cervical cancer burden [3].

The higher incidence and mortality rates of cervical cancer in LMICs such as Jamaica have been attributed to the low rates of cervical cancer screening [2]. Results from the Jamaica Health and Lifestyle Survey III 2016–2017 [4] found that approximately 70% of women aged 15–64 years reported ever receiving a Papanicolaou cytology screening (referred to as a Pap test). Furthermore, less than 50% of women of reproductive age (15–54 years) reported receiving a Pap test within the past three years [4]. These low rates of screening are concerning given findings from 2009 and 2013 which indicate a high prevalence of oncogenic Human Papilloma Virus (HPV) strains among Jamaican women [5, 6]. Hence, the Jamaican Ministry of Health and Wellness (MOHW) consider all women aged 21–65 years to be at risk for cervical cancer through sexual intercourse and recommend that they be screened every 3 years [7, 8]. The Pap test is the only screening test available in the Jamaican public health system and is free of cost. It is recommended that individuals at high risk for cervical cancer such as women and transgender men (who retain a cervix) with HIV infection should be screened annually beginning at the time of HIV diagnosis even if less than 21 years [7]. The Jamaica Cancer Society follows the MOHW guidelines and offers the Pap test at fixed sites and through mobile screening units [9]. The cost for the Pap test in the private sector ranges from J$2500 to J$5000 (US$17.60 to US$35.20). Increased and consistent uptake of Pap tests and better HPV vaccination coverage have largely been credited for the reductions in incidence and mortality of cervical cancer within high-income countries [10–12]. Similar reductions might be possible in Jamaica with adherence to the MOHW recommended Pap test screening guidelines and introduction of HPV testing into the public health system.

Previous research on cervical cancer screening uptake in Jamaica identified many barriers to screening. These ranged from sociodemographic factors, such as distance from screening locations, frequency of healthcare visits, and educational status of the women, to more personal factors, such as attitudes towards and perceptions of screening, reluctance to taking the Pap test, lack of awareness about screening facilities, lack of appropriate knowledge of cervical cancer, fear of the Pap test, perception that cervical screening is useful only for diagnosis of cancer and perception that they are not at risk [13–17]. Further, institutional factors, such as, the passive approach to cervical screening in Jamaica, routine screening of women only at postnatal visits and at the discretion of the healthcare provider, and long turn-around times for receiving test results have also been identified as barriers to cervical screening [17]. The present study was conducted to assess sociodemographic factors, attitudes, and knowledge associated with inappropriate adherence to Pap test screening guidelines and develop a model to estimate odds of Pap test screening among women living in western Jamaica.

## Methods

### Procedure

**Fig. 1:**
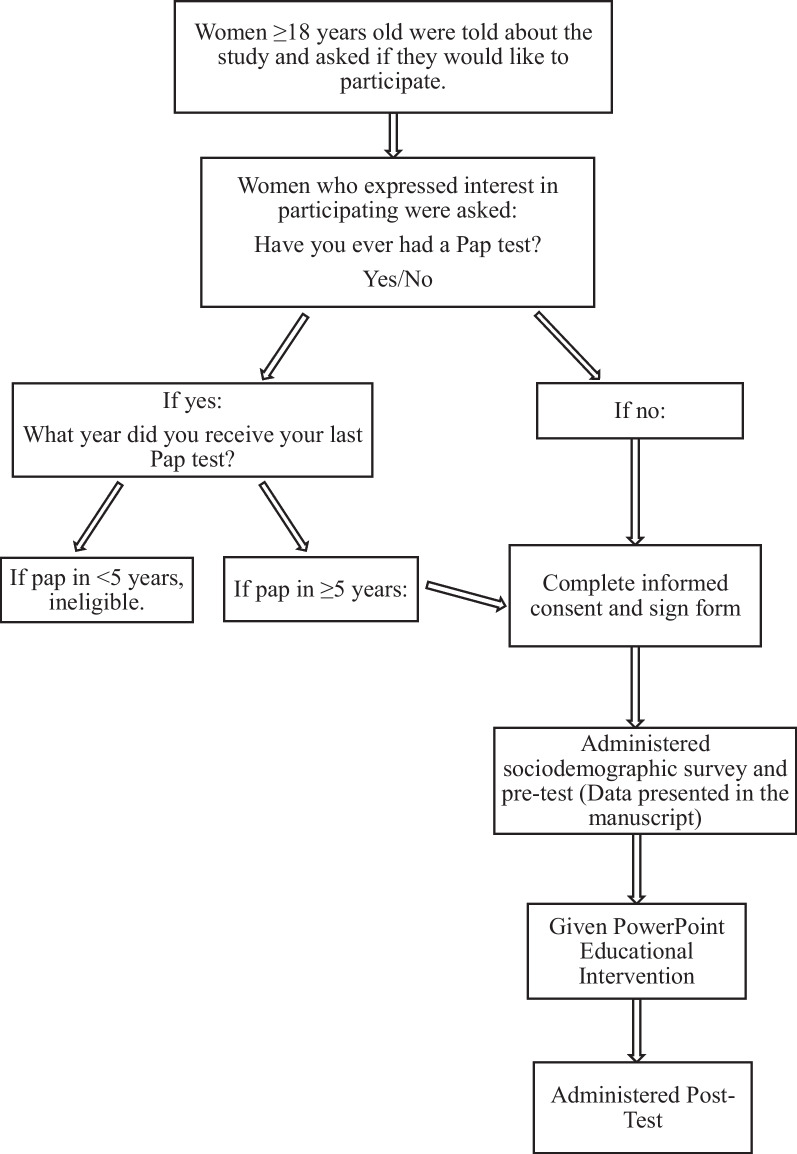
Outline of study procedures

In this cross-sectional study, we utilized sociodemographic and pre-test data that were collected from a convenience sample of 223 women between June and August of 2013 as part of an educational intervention designed to encourage uptake of cervical cancer screening (Fig. 1: Diagram outlining study procedures). Women attending health facilities and at community events in the four parishes of western Jamaica served by Jamaica’s Western Regional Health Authority (WRHA), namely St. James, Westmoreland, Trelawny, and Hanover, were recruited for the study [16]. These parishes provide no-cost Pap tests at hospitals and health centers in the parishes. To be eligible for the study women had to be age 18 years and older and had to have never had a Pap test or had not received a Pap test in the past five years or more. Potential participants were approached by the research team with information about the study. If a woman expressed interest in the study, she was asked “Have you ever had a Pap test?” Women who answered “No” were eligible to participate in the study. Women who answered “Yes” were asked to specify the year in which they last received a Pap test. If the date of the Pap test was five years or greater, they were eligible to participate. Women who received a Pap test in less than five years were excluded from the study.

Women who met the eligibility criteria were taken through the informed consent process by the research staff and encouraged to ask questions, after which signed informed consent was obtained.

The complete intervention study consisted of: (1) a sociodemographic survey with a pretest that assessed knowledge of (causes, symptoms) and attitudes about cervical cancer and cervical cancer screening; (2) a PowerPoint cervical cancer educational intervention; and (3) a posttest designed to assess changes in knowledge and attitudes about cervical cancer and cervical cancer screening [16]. The study procedures are outlined in Fig. 1. Only the demographic and pre-test data are presented in this paper. Data on the intervention and change in knowledge and attitude from the pretest to the posttest are presented in the manuscript by Coronado Interis et al. [16].

Although these data were collected in 2013, they are still currently relevant since the screening method and screening experience for women in Jamaica have not changed since 2013 [7, 8]. The Pap test is the only screening method that was, and is, available in the public health system. Although the 2020 Ministry of Health and Wellness Screening Guidelines lists HPV testing as a cervical screening method [7], HPV testing has not been implemented in the public health system and screening is still conducted solely by the Pap test, similar to what is stated in the 2011 Ministry of Health Jamaica National Guidelines for Cervical Cancer Prevention and Control [8].

### Primary outcome and predictor variables

The primary outcome of the analyses was previously receiving a Pap test. Predictor variables included sociodemographic characteristics, and knowledge and attitudes about Pap tests.

The following sociodemographic characteristics were analyzed: parish, age, highest education level, employment status, occupation, marital status, number of children, need for childcare, and distance from the nearest clinic. Age was categorized as 18–29, 30–39, 40–49, and 50 years or older. Highest education level was categorized as primary or less, secondary, and college, technical, vocational, or graduate school. Employment status was categorized as unemployed and employed. Occupation was categorized as unskilled worker, and skilled worker/professional/business owner. Marital status was categorized as married, single, cohabitating with partner, and other (divorced, separated, or widowed). Number of children was categorized as 0, 1, and 2 or more. Distance from clinic was categorized as ≤ 30 min and more than 30 min.

Attitudes included: (1) belief that the Pap test is embarrassing, and (2) belief that the Pap test is painful; responses were categorized as yes, no, or do not know. Additional questions assessed whether the women had heard of cervical cancer, their awareness of screening location and the purpose of the Pap test. Responses to these questions were categorized as yes, no, or do not know. To investigate sources of cervical cancer information, participants were asked whether they had heard of cervical screening from healthcare providers, the media, or other sources such as family, friends, or sexual partners to each of which they answered yes or no.

A knowledge score was constructed by summing correct answers from the 21-item pretest knowledge assessment with possible scores ranging from 0 to 21 (Table 1). The test assessed the following areas: knowledge of how women became infected with the agent that causes cervical cancer, symptoms of cervical cancer, and ways to prevent cervical cancer. After scores were created for all participants, tertiles were generated based on the score distribution and were used to categorize knowledge of cervical cancer as low, medium, and high.

**Table 1:** Questions used to generate knowledge score

*Section 1: How can people become infected with what causes cervical cancer?*	
Sexual intercourse	Yes/No/Not Sure
Skin contact genitals	Yes/No/Not Sure
Kissing	Yes/No/Not Sure
Witchcraft	Yes/No/Not Sure
Unsafe water/food	Yes/No/Not Sure
*Section 2: Which of the following symptoms would make you suspect you could have cervical cancer?*	
Bleeding after sexual intercourse	Yes/No/Not Sure
Bleeding in between menstrual cycles	Yes/No/Not Sure
Bleeding after menopause	Yes/No/Not Sure
Pain or burning sensation when peeing	Yes/No/Not Sure
Blood in vaginal discharge	Yes/No/Not Sure
Painful sexual intercourse	Yes/No/Not Sure
Pain in pelvis	Yes/No/Not Sure
*Section 3: Women can protect themselves from getting cervical cancer by:*	
Getting a Pap test	Yes/No/Not Sure
Using condoms	Yes/No/Not Sure
Being faithful to one sexual partner	Yes/No/Not Sure
Delaying having sex until after age 16	Yes/No/Not Sure
Avoiding smoking	Yes/No/Not Sure
Getting the HPV vaccine	Yes/No/Not Sure
*Section 4: Additional Questions*	
Cervical cancer is a preventable disease	Yes/No/Not Sure
According to Jamaica Cancer Society recommendations, how often should women get screened for cervical cancer?	□ Don’t’ know
	□ More than once a year
	□ Once a year
	□ Once every 2 or more years
	□ Only if they have symptoms
What is the purpose of a Pap test? Please choose ALL that apply:	□ Don’t know
	□ To check if a woman is pregnant
	□ To prevent cancer
	□ To diagnose sexually transmitted diseases
	□ To detect cancer
	□ Other (please specify)

### Statistical analysis

To assess associations between predictor variables and previous Pap test, chi-square tests and Fisher’s exact tests were conducted. A *p*-value of ≤ 0.05 threshold was used to indicate statistical significance. Crude and adjusted odds ratios and 95% confidence intervals were generated as measures of association for variables significant at the bivariate level and two other variables of interest (heard about screening from healthcare provider and knowledge about cervical cancer) and the outcome of ever having a Pap test. Participants with missing data were included in bivariate analyses but removed from the logistic regression model. All analyses were computed among complete cases and were conducted utilizing Statistical Analysis System (SAS) 9.4 (Cary, North Carolina).

## Results

Participant characteristics are described in Table 2. One hundred and nine women (48.9%) reported that they had received at least one Pap test previously. Significant differences in previous Pap test were found by parish, age group, employment status, and number of children. The proportion of participants from the parishes of Trelawny and St. James who had had a previous Pap exceeded 50%; 55.7% for St. James and 61.3% for Trelawny. Approximately 45.4% of participants from Westmoreland and 34.0% from Hanover reported previous Pap test.

**Table 2:** Participants characteristics by Pap test history ( *N* = 223)^a^

	Ever had a Pap test		*p*-Value^b^
	Yes	No	
	*N* = 109 (48.9%)	*N* = 114 (51.1%)	
Parish			**0.048**
Hanover	16 (34.0)	31 (66.0)	
St. James	44 (55.7)	35 (44.3)	
Trelawny	19 (61.3)	12 (38.7)	
Westmoreland	30 (45.4)	36 (54.6)	
Age (years)			** < 0.001**
18–29	18 (20.2)	71 (79.8)	
30–39	23 (67.7)	11 (32.3)	
40–49	22 (57.9)	16 (42.1)	
50 and older	46 (74.2)	16 (25.8)	
Highest education level			0.404
Primary or less	32 (55.2)	26 (44.8)	
Secondary	56 (47.9)	61 (52.1)	
College/technical/vocational/graduate	18 (41.9)	25 (58.1)	
Employment status			**0.003**
Employed	57 (60.6)	37 (39.4)	
Unemployed	49 (40.5)	72 (59.5)	
Occupation			0.64
Unskilled Worker	28 (58.3)	20 (41.7)	
Skilled worker/professional/business owner	29 (63.0)	17 (37.0)	
Marital Status			0.287
Single	65 (47.1)	73 (52.9)	
Married	19 (63.3)	11 (36.7)	
Cohabitating with Partner	19 (43.2)	25 (56.8)	
Other^c^	6 (60.0)	4 (40.0)	
Number of children			** < 0.001**
0	7 (12.7)	48 (87.3)	
1	19 (45.2)	23 (54.8)	
2 or more	83 (66.9)	41 (33.1)	
Childcare needed while screening			0.055
Yes	65 (55.1)	53 (44.9)	
No	43 (42.2)	59 (57.8)	
Distance from the clinic			0.273
30 min away or less	70 (46.4)	81 (53.6)	
More than 30 min away	38 (54.3)	32 (45.7)	

^a^Numbers may not always sum to total due to missing observations

^b^*P*-values significant at the 0.05 threshold are bolded

^c^Includes: separated, divorced, and widowed

The proportion of women in the youngest age group (18–29 years) who had previously been screened was low (~ 20%). The other age groups exceeded 50% screening, with the highest proportion of women who reported having had a Pap test (74.2%) in the oldest age group (50 years or older). The proportion of employed women who had been screened was 60.6% compared to 40.5% of unemployed women. A large proportion of women with no children (87.3%) reported not having been screened compared to 54.8% of women with one child and 33.1% of women with two or more children.

Participants’ attitudes and knowledge about Pap tests and cervical cancer are shown in Table 3. Significant differences in previous versus no previous Pap tests were found by participants’ belief that Pap tests were painful and for awareness of the purpose of Pap tests. The proportion of participants who had previously been screened was lower among those who thought that Pap tests were painful (40.0%) compared to those who did not think, or did not know, if Pap tests were painful (60.2%). The proportion of participants who had previously been screened was higher for those who knew the purpose of the Pap test (54.9%) compared to those who did not know its purpose (31.2%).

**Table 3:** Participant attitudes, knowledge and sources of information on cervical screening by previous Pap test (*N* = 223)^a^

	Ever had a Pap test		*p*-Value^b^
	Yes (109) (48.9%)	No (114) (51.1%)	
Believes Pap test is painful (*N* = 218, 97.8%)			**0.003**
Yes	48 (40.0)	72 (60.0)	
No/not sure	59 (60.2)	39 (39.8)	
Believes Pap test is embarrassing (*N* = 216, 96.9%)			0.538
Yes	10 (55.6)	8 (44.4)	
No/not sure	95 (48.0)	103 (52.0)	
Heard of cervical cancer (*N* = 222, 99.6%)			0.277
Yes	76 (51.7)	71 (48.3)	
No	33 (44.0)	42 (56.0)	
Aware of screening location (*N* = 218, 97.8%)			0.668
Yes	34 (46.6)	39 (53.4)	
No	72 (49.7)	73 (50.3)	
Aware of Pap test purpose (*N* = 217, 97.3%)			**0.001**
Yes	84 (54.9)	69 (45.1)	
No	20 (31.2)	44 (68.8)	
Heard about screening from healthcare provider (*N* = 218, 97.8%)			0.187
Yes	52 (53.6)	45 (46.4)	
No	54 (44.6)	67 (55.4)	
Heard about screening from media (*N* = 218, 97.8%)			0.996
Yes	36 (48.7)	38 (51.3)	
No	70 (48.6)	74 (51.4)	
Heard about screening from other^c^ sources (*N* = 218, 97.8%)			0.165
Yes	26 (41.3)	37 (58.7)	
No	80 (51.6)	75 (48.4)	
Knowledge of cervical cancer index score			
Score (*N* = 223, 100%)			0.293
Low	31 (41.9)	43 (58.1)	
Medium	33 (55.0)	27 (45.0)	
High	45 (50.6)	44 (49.4)	

^a^Numbers may not always sum to total due to missing observations

^b^*p*-Values significant at the 0.05 threshold are bolded

^c^Other sources include: family, friends, and sexual/romantic partners

The results of the crude and adjusted multivariable logistic regression analyses are presented in Table 4. The odds of having had prior Pap testing for women residing in the parish of St. James were more than 3 times the odds for women residing in the parish of Hanover (OR: 3.35; 95% CI: 1.12, 9.99). Similarly, the odds of having had prior Pap testing among women in Trelawny were more than 5 times the odds for women in Hanover (OR: 5.34; 95% CI: 1.23, 23.25), and women in Westmoreland had almost 4 times the odds as women in Hanover (OR: 3.70; 95% CI: 1.10, 12.50). Compared to women aged 18–29 years, the odds of having a previous Pap test among women who were 50 years and older were more than 6 times higher (OR: 6.17; 95% CI: 1.76, 21.54). The odds of previous Pap testing among employed women were 2.44 times higher than the odds among unemployed women (95% CI: 1.15, 5.20). Compared to women with no children, the odds of having a previous Pap test among women with one child were 4.15 higher (95% CI: 1.06, 16.22) and 8.43 times higher among women with two or more children (95% CI: 2.24, 31.63).

**Table 4:** Odds ratio of cervical screening by participants’ characteristics, attitude, and awareness of the Pap test

	Crude odds ratio (95% CI)^b^	Adjusted odds ratio (95% CI)^a,b^
Parish		
Hanover	Reference	Reference
St. James	**2.44 (1.15–5.15)**	**3.35 (1.12–9.99)**
Trelawny	**3.07 (1.12–7.86)**	**5.34 (1.23–23.25)**
Westmoreland	1.61 (0.75–3.50)	**3.70 (1.10–12.50)**
Age (years)		
18–29	Reference	Reference
30–39	**8.25 (3.40–19.99)**	3.05 (0.84–11.00)
40–49	**5.42 (2.37–12.39)**	3.09 (0.85–11.18)
50 and older	**11.34 (5.26–24.46)**	**6.17 (1.76–21.54)**
Employment status		
Employed	**2.26 (1.31–3.93)**	**2.44 (1.15–5.20)**
Unemployed	Reference	Reference
Number of children		
0	Reference	Reference
1	**5.67 (2.09–15.38)**	**4.15 (1.06–16.22)**
2 or more	**13.88 (5.78–33.36)**	**8.43 (2.24–31.63)**
Believe Pap test is painful		
Yes	**0.44 (0.26–0.76)**	**0.33 (0.16–0.71)**
No/Not Sure	Reference	Reference
Aware of Pap test purpose		
Yes	**2.68 (1.45–4.96)**	**3.90 (1.55–9.82)**
No	Reference	Reference
Heard about screening from healthcare provider		
Yes	1.43 (0.84–2.45	1.27 (0.55–2.93)
No	Reference	Reference
Knowledge about cervical cancer		
Low	Reference	Reference
Medium	1.70 (0.85–3.37)	0.99 (0.36–2.67)
High	1.42 (0.76–2.64)	0.91 (0.37–2.28)

*CI* confidence interval

^a^Adjusted for other variables included in table; ^b^ORs with CIs that are bolded do not include 1 and are considered statistically significant

Women who believed Pap tests were painful had 67% lower odds of having had prior Pap testing compared to women who did not think or were not sure that Pap tests were painful (95% CI: 0.16, 0.71). The odds of having had previous Pap testing among women who knew the purpose of Pap tests were almost 4 times the odds as among women who did not know the purpose of Pap tests (OR: 3.9; 95% CI: 1.55, 9.82).

## Discussion

Approximately 49% of the women in this study had previously been screened for cervical cancer with a Pap test but this test was conducted five years or more previously. This percentage is lower than the 70% reported from a national sample [4], and the 66% reported for a sample of women from the Northeastern parish of Portland [13]. Reports by Figueroa et al. 2005 and 1999 show that there has been no significant change in cervical cancer screening in Jamaica from 1993 when 40% of women were screened to 2000 when 36% were screened [18, 19]. Further, there has been no change in screening method from 2013 to the present time in Jamaica with the Pap test being the only screening test conducted in the public health sector [7, 8]. None of the women in this study met the Jamaican MOHW screening guideline recommendation of Pap test screening every three years.

Several sociodemographic, attitudinal, and cervical cancer awareness factors (parish, age, employment status, number of children, belief that Pap tests were painful, and awareness of the purpose of Pap tests) were found to be associated with previous Pap test screening among the women. Women in the parishes of St. James, Trelawny, and Westmoreland were more likely to have had a Pap test than women in the parish of Hanover. To our knowledge, this is the first study that compares cervical cancer screening by parishes in the western region of Jamaica.

Hanover is the smallest parish in the western region and the Noel Holmes General Hospital in the parish is a Type C hospital that provides primary care and basic secondary care services. Pap test samples are taken at the hospitals in Hanover, Trelawny (Type C hospital) and Savanna La Mar (Type B hospital) and sent to the Cornwall Regional Hospital (CRH) laboratory for processing. The CRH is the only Type A hospital, and the main hospital, in the region. Type A hospitals are multidisciplinary facilities that provide comprehensive secondary and tertiary health care services for all medical specialties and are referral centers for hospitals both in the public and private health systems. Type B hospitals provide primary and secondary care. Each parish in the region is given and manages its own budget based on the money available in the WRHA budget obtained from the Jamaican MOHW. Human resources (doctors, nurses, etc.) are established based on the size of population in each parish. Hanover, like other parishes, suffers from shortage of nurses and other health personnel that may help to account for the low rate of cervical screening. However, there seem to be other parish-specific barriers to cervical screening that should be investigated in future research so that they can be addressed.

Women aged 50 years and older were more likely to have received a Pap test than women aged 18–29 years. These findings are consistent with studies conducted in Jamaica and other LMICs that have found an association between older age and cervical cancer screening [13, 20, 21]. It is likely that older women have higher odds of exposure to Pap test because they have visited clinics, especially post-natal clinics, over a longer period of time. At postnatal visits, they were more likely to be recommended Pap test screening. This seems to be supported by our finding that women with one or two or more children had higher odds of previous Pap test than women with no children, similar to results from other research conducted in Jamaica and other LMICs [13, 22, 23]. Increased interaction with reproductive healthcare by pregnant women is a plausible explanation for their higher likelihood of being screened. In this study, the odds of screening also increased with increase in the number of children, from one to two or more. Therefore, it is essential that greater effort be made to target younger women who are sexually active but who are not yet accessing prenatal and postnatal services. This is even more urgent considering the high rates of infection with both high- and low-risk HPV strains among Jamaican women [5, 6] and the finding of an association between younger age and the occurrence of high-risk oncogenic HPV in one of the studies [6].

Women who were employed were more likely to receive Pap test screening than those who were unemployed. Unemployment has been associated with a lower likelihood of screening in other studies as well [22, 24, 25]. Employed women may have the financial resources to pay for Pap screening at private health facilities. Also, employers may promote screening of employees who may have health insurance coverage and access to both private and public health services. Additionally, public health departments sometimes visit companies and offices to provide mass screening as part of the Jamaican Ministry of Health and Wellness’ “wellness program.” Service in the government public health system in Jamaica as in many LMICs involves long waiting time and longer turnaround time for results. These factors help to deter uptake of cervical screening by women especially if the women do not encounter any cervical problems.

In contrast to the results of prior studies, we did not observe a significant relationship between knowledge of cervical cancer and previous cervical cancer screening [26, 27]. Possible explanations for this finding are that the women may know of cervical cancer but may not know of places where they can be tested, may consider the Pap test embarrassing, or may fear the test as they may think it is painful. Additionally, women may not know or understand that screening is necessary to identify pre-cancerous lesions so that the development of cervical cancer can be prevented. Some women believe that the test is conducted solely to diagnose cancer and fear finding out that they may have cancer. These reasons have been documented in other studies in Jamaica [13, 14, 21]. In this study, there was an inverse association between the belief that Pap tests are painful and previous Pap test screening. Fear of pain is a well-documented barrier to Pap test uptake for women in Jamaica as well as other countries [13, 14, 21, 27, 28]. Educational interventions to dispel these fears and misconceptions about the Pap test need to be conducted to increase uptake of screening.

Awareness that the purpose of the Pap test was to screen and treat pre-cancerous lesions to prevent cervical cancer was associated with increased odds of previous Pap test among the women. Thus, a major focus of cervical cancer campaigns and interventions in Jamaica should be to increase awareness of the benefit of screening in early identification of treatable pre-cancer lesions versus the consequences of not screening and allowing pre-cancer to progress to cancer. Previously, we found that women who knew the consequences of not receiving Pap test screening were more likely to be screened than those who reported that there were “no consequences” of not screening [13]. Therefore, there is a need to educate women on the benefit of screening as a cancer preventive method rather than as a method of diagnosing cancer. There are limitations to this study that should be considered in interpreting the results. Foremost is that the data were obtained from a convenience sample of women attending health facilities and community events in western Jamaica. All data were self-reported and subject to social desirability bias and recall bias; we did not have access to participant medical records and as such had no way of validating the reported previous Pap test. Lastly, not all participants answered every question, limiting the data available for each analysis.

## Conclusions

Despite its limitations, this study provided significant useful information on cervical cancer screening among women in the parishes of western Jamaica. The findings suggest that despite the availability of no-cost Pap tests in public health facilities, cervical cancer screening uptake in these parishes remains suboptimal. This highlights a need for urgent culturally appropriate and evidence-based interventions to engage women in cervical cancer screening. Younger women and women without children, in particular, could benefit most from interventions that engage them in general reproductive health care. Our findings of the differences in screening among women in the different parishes suggest that future research needs to be conducted to investigate and identify parish-specific barriers to cervical cancer screening that exist so that they may be addressed. Additionally, interventions designed to increase awareness that the purpose of the Pap test is to screen and treat pre-cancer to prevent cervical cancer is strongly warranted to promote screening, result in early treatment, and ultimately reduce the burden of cervical cancer among women in the western, and other regions, of Jamaica.

## Data Availability

The dataset used and analyzed during the current study are available from the corresponding author upon reasonable request.
